# Walking activity increases physical abilities and subjective health in people with seven different types of disabilities

**DOI:** 10.3389/fpubh.2023.1120926

**Published:** 2023-06-16

**Authors:** Pattamon Selanon, Warawoot Chuangchai

**Affiliations:** Thammasat University Research Unit in Making of Place and Landscape, Faculty of Architecture and Planning, Thammasat University, Pathum Thani, Thailand

**Keywords:** gait, physical activity, physical performance, inclusive population, person with special needs

## Abstract

**Introduction:**

People with disabilities have a great risk of physical inactivity, which causes several diseases, dependency, and long-term care. Walking helps to increase physical activity, which leads to better overall health and independence. However, less research attention has focused on walking for people with disabilities, and even fewer studies have been considered for different types of disabilities. The present study aimed to demonstrate how walking distance was associated with people with seven different types of disabilities— including visual, hearing, physical/mobility, intellectual, learning, autism, and emotional/behavioral disabilities—in terms of their physical abilities and subjective health.

**Methods:**

A total of 378 participants (aged 13–65) were gathered from seven national organizations in Thailand. A survey questionnaire on aspects of physical abilities (i.e., walking distance or manually rolling wheelchair distance; body balance; weightlifting; exercise duration and frequency); and subjective health (i.e., health status and satisfaction) was completed online by all participants.

**Results:**

The walking distance was partially positive and associated with exercise duration, weightlifting, exercise frequency, and health status (all p values < 0.001), as well as body balance and health satisfaction (p = 0.001 and 0.004, respectively), after controlling for age, sex, and types of disability. This demonstrated that increasing the amount of distance walked could well lead to a more positive body and mind.

**Discussion:**

The present study suggests that the possibility of having a walk and/or encouraging people with disabilities to walk for greater distances can have a significant impact on both their physical and subjective health outcomes.

## 1. Introduction

An important goal of public health promotion across the world is to acquire strategies to enhance physical activity and eliminate sedentary time in order to improve population health and decrease the occurrence of a number of preventable illnesses and impairments (1, 2). Evidence of the poor health and health behaviors of people with disabilities has established the fact that encouraging greater physical activity and far less sedentary behavior is a key focus for eliminating health inequities (3). One of the most successful physical activities is walking, which is also the first thing most people decide to do as a starting point for beginning exercise (4, 5). Walking is a sustainable activity that could simply be incorporated into everyday life to enhance both physical (e.g., postural stability, motor control, and muscular strength and endurance) and mental health (e.g., emotional well-being and satisfaction) (6, 7). Walking could also support the ability to do daily activities, promote independent living, and progressively improve the quality of life for all people (8, 9). Thus, walking more can be a great, practical solution that can make a significant change in the health of all people, including people with disabilities.

Walking is a normal action that requires coordination across several bodily systems and becomes more difficult over time as the body ages and naturally deteriorates (10). More challenges occur in people with disabilities since they lose fully or partially part of one or more functions to perform a normal walk (11). Although each of the seven different types of disabilities has unique characteristics, some may also share certain similarities. People with sensory (visual and hearing) and physical/mobility disabilities are typically restricted in mobility since they rely on support from assistive care or devices, such as a cane, crutch, walker, or wheelchair (12–14). People with intellectual and learning disabilities experience gait abnormalities or delays, which are caused by a combination of cognitive and motor coordination impairments (11, 15). Due to poor social skills and unpredictable or uncontrollable behaviors, people with autism and emotional/behavioral disabilities tend to struggle when participating in outdoor activities (16, 17).

Subjective health, also known as subjective well-being or self-rated health, represents an individual’s overall sense or self-perceived perception of their current level of well-being (18). Since each individual is subjectively influenced by the degree of disability (including diseases that cause disability), the subjective health index (e.g., health status and satisfaction) has become an important indicator that is widely used in gerontology, illness, and disability investigations (19, 20). Self-ratings of health status and satisfaction have been studied across both non-disability and disability populations (21–23), which found that people with disabilities were more likely to assess themselves lower than their non-disabled peers (24, 25). Chronic diseases, illnesses, and pains were found to have a major negative impact on health status and satisfaction for people with disabilities in particular (26–28). Within the group of people with sensory impairments, the lowest rate of health status was reported by people with both visual and auditory impairments compared to either people with only visual impairment or only auditory impairment (29). A population-based study in China suggested the adoption of subjective health as a national screening method for survey research in the field of public health (30). Also, similar uses have been found in a number of previous studies, including a cross-national study in children and adolescents (31), in various health-related status and satisfaction questions (32), and among patients for clinical purposes (33).

Globally, people experiencing disability, whether permanently or temporarily, number over a billion (34). An inclusive population is increasing every year since the world population is aging rapidly (35), especially in developing countries where there are generally unmet healthcare needs (34). People with disabilities reportedly have a higher rate of physical inactivity and a greater prevalence of noncommunicable diseases (NCDs), such as obesity, diabetes, and cardiovascular disease, than those without disabilities (36, 37). Physical activity studies among people with disabilities are more important and require much more research attention (38). However, there has been less research into the associations between walking distance and health benefits specifically for people with disabilities, and very few studies have focused on more than three different types of disabilities. The present study aims to investigate the correlations between walking distance and health benefits across all seven types of disabilities, based on their physical abilities and subjective health. By addressing the knowledge gap, the present study will contribute to a better understanding of physical and health characteristics as well as the underlying connections related to walking as a physical activity for an inclusive population.

## 2. Materials and methods

### 2.1. Participants

The present study was undertaken with the assistance of seven national organizations, including the Thailand Association of the Blind, the National Association of the Deaf in Thailand, the Association of Persons with Physical Disability International, the Association of Persons with Intelligence Disability of Thailand, the Thai Association to Encourage the Potential of Learning Disabled Persons, the Association for the Mentally Ill of Thailand, and the Autistic Thai Foundation. All prospective candidates who were members of one of those organizations were invited to the initial screening by the official administrator of each organization. Male and female individuals who had been diagnosed by medical professionals with visual, hearing, physical/mobility, intellectual, learning, autism, or emotional/behavioral disabilities, between the ages of 13 and 65, and could perform daily self-care activities were included. However, those who had such severe immobility (e.g., bedridden), were unable to be contacted by their organizations or the research team during the study or were unable to understand the main objectives of the study were excluded. The sample size calculation was estimated using both the correlation analysis guideline (for each type of disability) and the population guideline (for the total sample) to be able to detect a power of 80% (39, 40). A 25% dropout rate was incorporated into the estimation to prevent attrition bias. Consequently, the present study required a minimum of 54 participants per type of disability.

**Figure 1:**
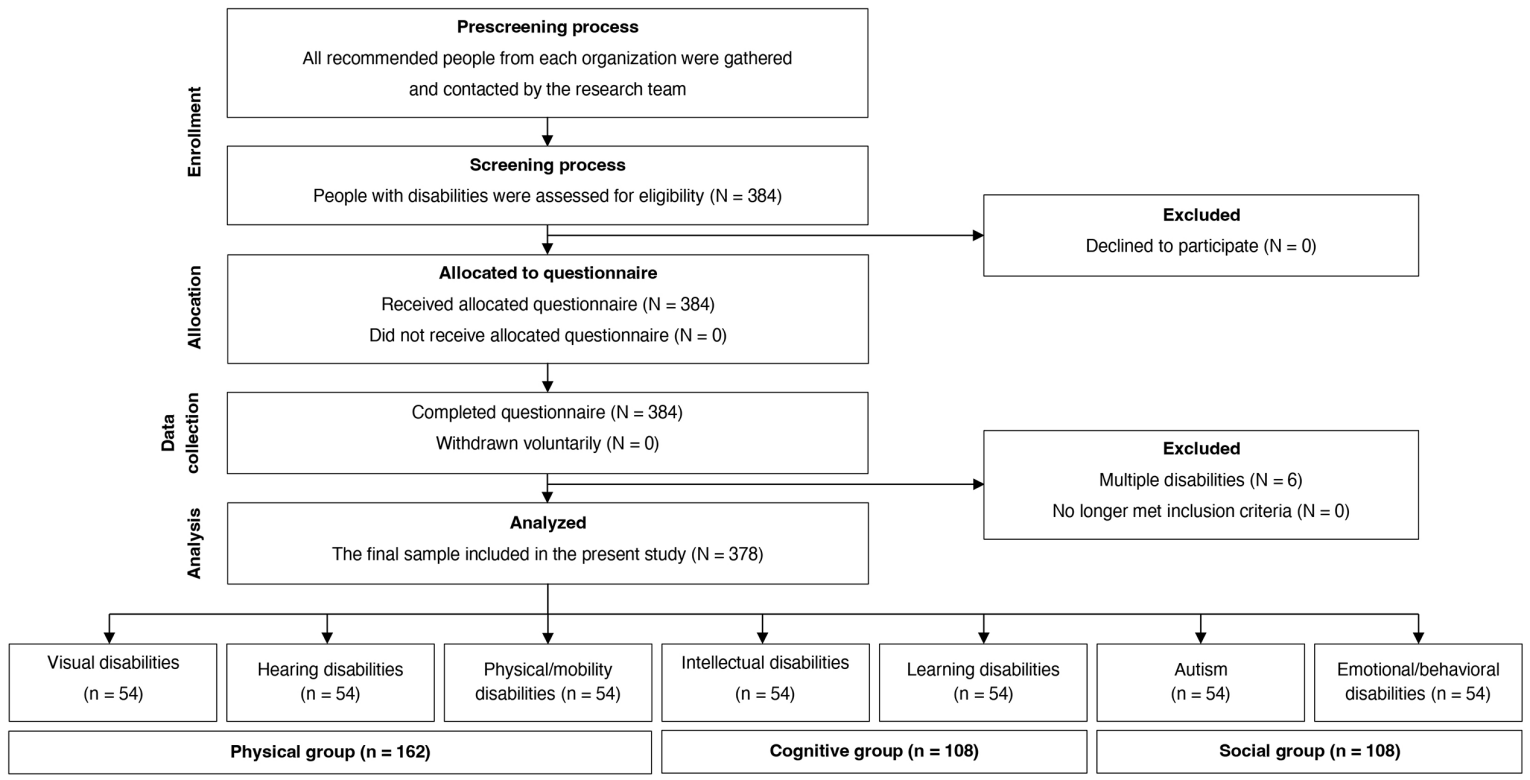
A flow diagram displaying the study sample.

At the preliminary stage, 384 individuals were qualified. Among them, 6 individuals were subsequently excluded since it was discovered that they had more than one type of disability (also known as multiple disabilities), resulting in a final sample size of 378 participants (54 from each of the seven types of disabilities). Once recruited, participants with visual, hearing, and physical/mobility disabilities were categorized as the physical group (*n* = 162), which found that all wheelchair participants were using manuals. Participants with intellectual and learning disabilities were characterized as the cognitive group (*n* = 108). Participants with autism and emotional/behavioral disabilities were referred to as the social group (*n* = 108), as shown in Figure 1. The present study was granted ethical approval by the Human Research Ethics Committee of Thammasat University: Social Sciences (Number 102/2564) and approved by the Thai Clinical Trials Registry Committee (TCTR20220806001). All participants and/or their parents or legal guardians received all the substantial information along with the survey questionnaire and gave their written, informed consent to participate in the present study prior to the measurement.

### 2.2. Measurements

The survey questionnaire, which had three main parts—personal information, physical ability, and subjective health—was specifically structured for people with disabilities by primarily using choice questions and rating scales.

The personal information part asked for details on age (in years), sex (male or female), occupation (studying, full-time and part-time working, or workless), place of residence (urban or suburban), and medical condition (having any NCDs or not; a list of NCDs was provided).

The physical ability part required the participants to select one of the given choices that best suited the question by evaluating themselves or recalling recent experiences. All questions were ranked by three levels. The distance covered by walking (or rolling on a manual wheelchair) was ranked as 50 m or less, more than 50 to 500 m, and over 500 m. For body balance, inability or difficulty in balancing was classified as low, dependence on an assistive device or person was classified as moderate, and independence in balancing was classified as high. Weightlifting was ranked as 3 kg or less, more than 3 to 5 kg, and over 5 kg. Exercise duration was ranked as less than 15, between 15 and less than 30 min, and 30 min or more, whereas frequency was ranked as monthly, weekly, and mostly every day.

The subjective health part was comprised of two self-rating scale questions, which were suggested by the literature (19, 31, 41). First, how would you feel about your overall health, apart from the disability? where 0 and 10 points indicate being unhealthy at all and very healthy, respectively. Second, how satisfied would you feel with your health? where 0 and 10 points indicate being unsatisfied at all and very satisfied, respectively.

The survey was carried out between November 2021 and January 2022. Due to the COVID-19 situation in Thailand, the participants were asked to complete the survey online (e.g., on a mobile phone, tablet, or computer). The research team offered personal support to participants who were experiencing trouble or feeling uncomfortable completing the survey through phone or video calls upon request. Each question was given without a time limit in order to protect participants from mental stress or fatigue that may be caused by answering the lengthy number of questions. During completing the survey, the participants were allowed to pause at any moment for an unlimited length of time as well.

### 2.3. Statistical analysis

Descriptive statistics of the participants, including personal data, physical abilities, and subjective health, were described either as numbers with percentages (%) or medians (interquartile range of 25th and 75th percentile) in Tables 1, 2. For nonparametric data, the Kruskal-Wallis test was used to compare the three groups, and the Mann–Whitney *U* test was then used for pairwise comparisons between any two of those three groups (Table 2). Eta-squared was calculated via the *z* value squared divided by the total sample and reported as an effect size (42) for the comparison results (Table 2). A partial Spearman rank correlation was employed to examine the relationships between the walking distance and all of the physical ability and subjective health variables. Correlation coefficients (*r*) for the entire sample (with age, sex, and seven different types of disabilities adjusted) and for each group (with age and sex adjusted) were examined (Table 3). The level of significance of *p* < 0.05 (two-tailed) was set as a minimum for all tests. All statistical analyses were performed using IBM SPSS Statistics, version 22.

**Table 1:** Participant characteristics: descriptive statistics, multiple comparison results, and pairwise comparison results.

Variable (Unit)	Total sample (*N* = 378)	Physical group (PG, *n* = 162)	Cognitive group (CG, *n* = 108)	Social group (SG, *n* = 108)	Multiple comparison results	Pairwise comparison results		
						PG-CG	CG-SG	PG-SG
Age (years)	33.00 (14.00, 60.00)	33.00 (14.00, 60.00)	30.00 (14.00, 60.00)	35.50 (14.00, 60.00)	NS	NS	NS	NS
Female	192 (50.80)	88 (54.30)	53 (49.10)	51 (47.20)	NS	NS	NS	NS
Occupation					*	NS	**	*
Student	132 (34.90)	57 (35.20)	36 (33.30)	39 (36.10)				
Employed	225 (59.50)	96 (59.30)	60 (55.60)	69 (63.90)				
Unemployed	21 (5.60)	9 (5.60)	12 (11.10)	0				
Urban resident	231 (61.10)	106 (65.40)	47 (43.50)	78 (72.20)	***	***	***	NS
No NCDs	276 (73.00)	130 (80.20)	77 (71.30)	69 (63.90)	*	NS	NS	**

Results are presented as median (interquartile range 25th, 75th percentile) or number (%). NCDs, noncommunicable diseases; PG, physical group; CG, cognitive group; SG, social group. NS, no significance; *, **, and *** denote significance levels at *p* < 0.05, *p* < 0.01, and *p* < 0.001, respectively.

**Table 2:** Physical abilities and subjective health: descriptive statistics, multiple comparison results, and pairwise comparison results.

Variable (Unit)	Total sample	Physical group (PG)	Cognitive group (CG)	Social group (SG)	Multiple comparison results	Pairwise comparison results		
						PG-CG	CG-SG	PG-SG
Walking distance (m)					***	**	NS	***
≤ 50	87 (23.00)	65 (40.10)	17 (15.70)	5 (4.60)				
> 50 to 500	82 (21.70)	23 (14.20)	26 (24.10)	33 (30.60)				
> 500	209 (55.30)	74 (45.70)	65 (60.20)	70 (64.80)				
Body balance (level)					***	**	NS	**
Low	9 (2.40)	9 (5.60)	0	0				
Moderate	14 (3.70)	10 (6.20)	2 (1.90)	2 (1.90)				
High	335 (93.90)	143 (88.30)	106 (98.10)	106 (98.10)				
Weightlifting (kg)					**	**	**	NS
≤ 3	123 (32.50)	48 (29.60)	50 (46.30)	25 (23.10)				
> 3 to 5	122 (32.50)	49 (30.20)	30 (27.80)	43 (39.80)				
> 5	133 (35.20)	65 (40.10)	28 (25.90)	40 (37.00)				
Exercise duration (min)					***	***	*	NS
< 15	139 (36.80)	72 (44.40)	33 (30.60)	34 (31.50)				
15 to <30	111 (29.40)	51 (31.50)	18 (16.70)	42 (38.90)				
≥ 30	128 (33.90)	39 (24.10)	57 (52.80)	32 (29.60)				
Exercise frequency (level)					***	**	NS	***
Monthly	125 (33.10)	82 (50.60)	26 (24.10)	17 (15.70)				
Weekly	143 (37.80)	36 (22.20)	53 (49.10)	54 (50.00)				
Mostly every day	110 (29.10)	44 (27.20)	29 (26.90)	37 (34.30)				
Health status (10 points)	8.00 (7.00, 10.00)	8.00 (7.00, 10.00)	10.00 (8.00, 10.00)	8.00 (7.00, 10.00)	**	**	*	NS
Health satisfaction (10 points)	10.00 (7.00, 10.00)	10.00 (7.00, 10.00)	10.00 (8.00, 10.00)	8.25 (6.00, 10.00)	***	**	***	**

Results are presented as number (%) or median (interquartile range 25th, 75th percentile). PG, physical group; CG, cognitive group; SG, social group; NS, no significance. ^*^, ^**^, and ^***^ denote significance levels at *p* < 0.05, *p* < 0.01, and *p* < 0.001, respectively.

**Table 3:** Partial rank correlations between walking distance and variables related to physical abilities and subjective health.

Variable (Unit)	Walking distance of							
	Total sample^a^		Physical group^b^		Cognitive group^b^		Social group^b^	
	*r*	*p* value	*r*	*p* value	*r*	*p* value	*r*	*p* value
Physical ability (level)								
Body balance	0.17	**	0.16	NS	0.18	NS	0.22	*
Weightlifting	0.31	***	0.07	NS	0.55	***	0.34	***
Exercise duration	0.67	***	0.73	***	0.71	***	0.55	***
Exercise frequency	0.26	***	0.22	**	0.39	***	0.11	NS
Subjective health (point)								
Health status	0.19	***	0.21	**	0.54	***	−0.24	*
Health satisfaction	0.15	**	0.38	***	0.38	***	−0.31	**

Characters^a^ and ^b^ indicate partial correlation results with age, sex, and seven types of disabilities adjusted and with age and sex adjusted, respectively. *r*, correlation coefficient, NS, no significance; *, **, and *** denote significance levels at *p* < 0.05, *p* < 0.01, and *p* < 0.001, respectively.

## 3. Results

The characteristics of all participants and each of the three groups are reported in Table 1. Overall, the present study had participants in a similar range of age (around 33 years) and sex proportion (50.80% of females), which supported the homogeneity of the sample. There were, however, some variations in occupation (*p* = 0.013), place of residence (*p* < 0.001), and health condition in NCDs (*p* = 0.011). In terms of occupation, 59.50% were employed, 34.90% were students, and 5.60% were unemployed, whereas the social group differed statistically significantly from the cognitive and physical groups (*p* = 0.002 and 0.030, respectively). Meanwhile, the cognitive group had the lowest number of members (47 individuals) who lived in Bangkok, which was a statistically significant difference to the physical and social groups (106 and 78 individuals, respectively, all *p* values <0.001). Even though there were more participants without NCDs in the physical group (130 people) than in the cognitive and social groups (77 and 69 people, respectively), only a statistically significant difference was found between the physical and social groups (*p* = 0.003).

The comparisons of physical abilities and subjective health across all groups are reported in Table 2. Health satisfaction was found to be the only one with a statistically significant difference in each group comparison (*p* values from less than 0.001 to 0.005 and eta-squared values from 0.02 to 0.09, indicating a low to high effect size). The multiple comparison results exhibited statistically significant differences in all physical abilities and in health status (*p* values from less than 0.001 to 0.007 and eta-squared values from 0.02 to 0.10, indicating a low to high effect size). A similar pattern was discovered in the comparison results between the physical and cognitive groups (*p* values from less than 0.001 to 0.004 and eta-squared values from 0.01 to 0.11, indicating a low to moderate effect size). The social group revealed statistically significant differences from the physical group in certain abilities related to walking distance, body balance, and exercise frequency (*p* values from less than 0.001 to 0.003 and eta-squared values from 0.01 to 0.07, indicating a low to high effect size). On the other hand, in weightlifting, exercise duration, and health status, there were statistically significant differences between the social and cognitive groups (*p* values from less than 0.002 to 0.025 and eta-squared values from 0.02 to 0.04, indicating a low to medium effect size).

The partial rank correlations for the entire sample (after controlling for age, sex, and seven types of disabilities) and for each group (after controlling for age and sex) are reported in Table 3. The results showed that for the total sample, the walking distance was positively and statistically significantly correlated with all physical abilities and subjective health (*p* values from less than 0.001 to 0.004 and *r* values from 0.15 to 0.67, indicating weak to moderate relationships). The walking distance showed positive and statistically significant correlations with all subjective health measures and some physical abilities (exercise duration and frequency) for the physical group (*p* values from less than 0.001 to 0.007 and *r* values from 0.21 to 0.73, indicating weak to strong relationships). For the cognitive group, the walking distance was positively and statistically significantly correlated with all physical abilities (except for body balance) and subjective health (all *p* values less than 0.001 and *r* values from 0.38 to 0.71, indicating weak to strong relationships). Meanwhile, the walking distance of the social group was statistically significant and negatively correlated with all subjective health (p values from less than 0.001 to 0.015 and *r* values from −0.24 to −0.31, indicating weak relationships) and positively correlated with all physical abilities, except exercise frequency (*p* values from less than 0.001 to 0.021 and *r* values from 0.22 to 0.55, indicating weak to moderate relationships). Thus, only the duration of exercise, health status, and health satisfaction were found to be statistically significantly correlated with walking distance across all groups.

## 4. Discussion

To the best of our knowledge, the present study is the first to investigate people with seven different types of disabilities in connection with walking distance as a physical activity through both their physical abilities and subjective health. For comparing the three groups, the present study found that physical ability (i.e., walking distance, body balance, weightlifting, exercise duration, and frequency) and subjective health (i.e., health status and satisfaction) were different in all of their sub variables. Meanwhile, the physical, cognitive, and social groups could be differentiated from one another by health satisfaction. In terms of correlations, walking distance in the total group was positively associated with all sub variables of physical ability (i.e., body balance, weightlifting, exercise duration, and frequency) and subjective health (i.e., health status and satisfaction). The walking distance was also associated with exercise duration, health status, and health satisfaction in a positive way across all groups, but it was associated with health status and satisfaction in a negative way only in the social group.

### 4.1. Interpretations

Our findings indicated that each group appeared to have an individualistic approach to physical activity participation, which was dependent on their physical abilities and/or on how they perceive their subjective health. The differences between the physical and cognitive groups were obvious, which may be impacted by their distinct living regions (urban and rural), aside from the varied types of disabilities. Another interesting finding was in the social group, which demonstrated all of the differences in physical abilities and subjective health but only some in the physical group and some in the cognitive group. According to our findings, the physical and cognitive groups may differ from the social group due to the various health problems caused by NCDs and the various regions where individuals resided, respectively. While age and gender did not indicate any differences regarding health satisfaction scores in earlier studies (23, 43), the findings varied among our three groups. Unlike the physical abilities, the subjective score of health satisfaction was the only measure that could be identified among the three groups in this investigation. Our health satisfaction findings were confirmed by a prior subjective health study, which indicated that even people with similar medical conditions or equivalent stages of impairment can have varying levels of self-perceived wellbeing since each person’s subjective and unique response is different (19). The social group had the lowest median score for health satisfaction, which was consistent with similar studies on people with autism spectrum disorder. When compared to their typical peers, people with autism self-reported being less satisfied with themselves and with their lives (44), having less perceived happiness (45), poor mental health with more anxiety symptoms (46), and negative healthcare experiences that were associated with triggers for anxiety, shutdowns, and meltdowns, all of which somehow contributed to their health satisfaction (47). This illustrated that when it comes to a physical activity context, people with disabilities seemed to perceive their satisfaction with their health differently, which suggested the use of the health satisfaction score in classifying the group differences in relation to the type of disabilities.

Our findings with all of the participants showed that the greater the walking distance acquired as a physical activity, the healthier everyone was in terms of both physical and emotional expressions. These were in agreement with numerous previous studies that have shown that physical activity, including walking, can improve people with disabilities in many aspects, both physically and mentally, as well as socially (48–51). A closer look at each group revealed that the cognitive group benefited slightly more from active walking—with only body balancing having a negligible effect—than the physical and social groups. This may be explained by the fact that some participants in the physical group used wheelchairs, which may have inhibited or limited their ability to effectively gain the benefits, whereas the social group appeared to have a difficult time with active walking because it was regularly performed in public areas, which may have been the barrier to gaining the full benefits. Active walking was recommended to be an effective intervention for the cognitive group, as suggested by previous studies on people with intellectual disabilities, since it was a lifestyle physical activity suitable for their daily routines (52, 53). For children, a dual-task exercise (combined with active walking) was proposed to improve cognitive health and performance (54). Aerobic exercise with a peer-guided program that incorporates active walking (on a treadmill) revealed that adolescents with intellectual disabilities enhanced their health-related physical fitness, as evaluated by curl-ups, a 6-min walk, and body mass index (55). However, some past studies on adults with intellectual disabilities observed no substantial health improvements (e.g., blood pressure or body composition) from walking activity and acknowledged that their participants required a more rigorous engagement to achieve those expected health benefits (2, 56).

Moreover, our findings supported the idea that the amount of walking distance that people with disabilities can cover reflects their physical abilities, which are related to the amount of exercise they can accomplish (2). Counting steps is another way to refer to walking distance. A research review proposed public health guidelines for step counting: an average of 1,200 to 8,800 steps per day was suitable for special populations (including people with disabilities), and an average of 2,000 to 9,000 steps per day was suitable for healthy older adults (8). For health status and satisfaction, our findings indicate mixed directions of relationships. This inconsistent pattern may be explained by the fact that people with disabilities, in general, feel better about their health when they can walk longer distances. However, people with autism and emotional/behavioral problems may experience the opposite in certain situations due to their specific characteristics (e.g., social skill deficits or aggression) (16, 57). On the other hand, by engaging in physical activity, including walking, children with autism were found to have an advantage in helping them better control their anger (58). On one aspect of leisure walking (1), prior research on natural areas suggested that experiences with nature (e.g., green spaces or public parks) offered emotional and social benefits to people with autism, mood, and behavioral disorders, as the environment served as a buffer zone for stress relief and positive emotions of relaxation and happiness. Nevertheless, the effects were insufficient to cure or prevent any of these disorders (59, 60). Therefore, our findings suggested that a more physically active walk, as indicated by a longer walking distance, had the potential to improve the physical abilities and subjective health as well as the overall health of people with disabilities. Alternatively, walking should be promoted as a form of active rehabilitation by health care practitioners and medical professionals, and health authorities and other relevant parties should discuss ways to more effectively incorporate walking into the planning and design of cities in a way that is safe, pleasant, and arranged by supported facilities (e.g., accessible public drinking water, resting seats, and restrooms) so that the benefits of walking can be realized by all members of society.

### 4.2. Implications for public health policies and practical recommendations

Encouraging people with disabilities to engage in walking activity as a health promotion strategy requires a multifaceted approach involving long-term collaboration between governments, public health agencies, and community organizations (1, 34). By implementing these public health policies and practical recommendations into action, walking activity among people with disabilities can occur and be successful, ultimately leading to the benefits being shared with all populations.

Create opportunities for walking activity programs: promoting opportunities for people with disabilities to walk should be prioritized by public health policies, which can be accomplished through programs such as walking groups, guided walks (or buddy systems), or campaigns for adaptive walking (52, 55). Government agencies can collaborate and partner with public health policies, disability organizations, and local community groups to develop and implement walking activity programs that are culturally appropriate and accessible to diverse populations by providing assistance and accommodations to overcome participation barriers in terms of adaptive equipment, accessible facilities, and trained staff (52). These corroborating aspects can also contribute to the formation of social support groups and networks as a key motivator that encourages walking through a sense of community (1).

Offer financial incentives: as an extension of the previous point, public health policies can provide incentives for individuals with disabilities to walk more. For instance, governments could offer free public transportation passes, tax incentives, health insurance discounts, or other financial rewards to individuals who consistently participate in walking programs or achieve certain objectives (61).

Increase public awareness and provide education: numerous people who have disabilities may be unaware of the advantages of walking, which is a low-cost, straightforward, and efficient form of physical activity (2). Governments can collaborate with public health initiatives to create awareness campaigns that can potentially shape people’s perceptions of walking by eliminating common misconceptions and providing details concerning the benefits of walking (34, 61). In addition, governments and public health programs should provide education and instruction on how to engage in safe walking activity that is suitable for the specific type of disability, as well as how to incorporate it as a regular physical activity into daily life (62).

Promote accessible environments and inclusive infrastructure: one of the major barriers to mobility for people with disabilities, particularly in developing countries, is the physical environment. It is important to make walking routes and public spaces accessible and safe (4, 62). This includes ensuring that, for instance, sidewalks are wide enough to accommodate wheelchairs (or mobility assistance) and that pedestrian crossings have audible signals for people with visual problems. Governments and public health policies can work together to stimulate the implementation of streetscape and transportation accessibility guidelines and standards. Additionally, governments should work collaboratively with private companies to implement accessibility elements such as ramps at public transportation hubs and destinations, as well as to develop accessible surroundings and services for public parks and recreational spaces, in order to minimize accidents and encourage people with disabilities to walk (4, 62). In terms of safety, technology can assist individuals in tracking their walks via mobile apps or portable devices (e.g., smart watches or fitness trackers) to alert them when their surroundings appear to require more caution, such as when it is dark or raining, when there are no path lights, or when there are construction sites nearby (4). On the other hand, individuals can be motivated to walk by allowing them to plan their own routes based on their subjective interests, providing walking reminders, and keeping them connected with local walking societies.

### 4.3. Strengths, limitations, and future directions

The present study successfully gathered participants with a diverse range of disabilities and a wide age range. A noninvasive measuring technique (the survey) was only used in the study, which was one of the safest ways to investigate an inclusive population without any health-related issues throughout the trial. Since our participants were referred to a sensitive group, the online survey provided them with a contactless option (particularly during the COVID-19 pandemic), which promoted honest responses as well as an opportunity to complete the survey freely at their own pace and in a convenient time and environment. Despite the success of the present study, there were some limitations. First, no people with multiple disabilities were analyzed since the sample size was relatively small and went beyond the scope of our study design. Second, there is a potential for inaccurate measurement from the surveys that are not completed by participants themselves (e.g., by their relatives, guardians, or representatives). Those outcomes are expected to be small, although future work should be mindfully carried out by taking this concern into account. Third, health-related data (e.g., body mass index, nutrition, or lifestyle), physical experiments (e.g., walk or postural stability tests), and physiological assessments (e.g., blood pressure, heart rate variability, cognitive or stress tests), all of which have been indicated to be associated with physical abilities, were not examined. Thus, the interpretation of the results must be made with caution. Importantly, while it was true that the choice questionnaire, which was mostly employed in the present study, offered various advantages, the ordinal or categorical data acquired from this method restricted the statistical analysis choices. Future research should include collecting data on interval and/or continuous scales for more statistical analysis alternatives (e.g., regression analysis), which can lead to not only improved outcomes but also a deeper level of discussion and comprehension. Further studies that include people with multiple disabilities and collect more objective data in terms of both physical and mental evaluations are recommended as well.

## 5. Conclusion

Seven different types of disabilities were recruited and completed the online survey. Based on characteristics, three groups were formed, namely, the physical (visual, hearing, and physical/mobility disabilities), cognitive (intellectual and learning disabilities), and social (autism and emotional/behavioral disabilities) groups. The sole indicator that could discriminate differences among those groups, as opposed to their physical abilities, was found to be health satisfaction. The walking distance was shown to have positive relationships with exercise duration, health status, and health satisfaction across all groups except the social group, where it had negative relationships with health status and satisfaction. On the other hand, by involving active walking that causes unpleasant feelings, it can also be considered a developmental process against sedentary activity for the social group. Therefore, the present study highlighted the need to encourage longer walks (or distances for non-electric wheelchairs), which would eventually lead to an increase in physical abilities and subjective health among people with disabilities. The importance of physical activity, such as active walking, in contributing to wellbeing across the lifespan is beneficial and should be encouraged by health-related providers to incorporate as a rehabilitation strategy and considered by health specialists and key stakeholders to improve environmental planning in supporting walking for an inclusive population.

## Data availability statement

The original contributions presented in the study are included in the article/supplementary material, further inquiries can be directed to the corresponding author: warawoot.chu@dome.tu.ac.th.

## Ethics statement

The studies involving human participants were reviewed and approved by the Human Research Ethics Committee of Thammasat University: Social Sciences (Number 102/2564). Written informed consent to participate in this study was provided by the participants’ legal guardian/next of kin.

## Author contributions

PS designed the survey questionnaire used in the study, performed the measurement, contributed to the data collection, and wrote and edited the manuscript. WC analyzed the data, interpreted the results, and drafted and revised the manuscript. All authors contributed to the article and approved the submitted version.

## Funding

This work received funding support from the NSRF via the Program Management Unit for Human Resources and Institutional Development, Research and Innovation [Grant number B05F640096] and was supported by the Thammasat Postdoctoral Fellowship.

## Conflict of interest

The authors declare that the research was conducted in the absence of any commercial or financial relationships that could be construed as a potential conflict of interest.

## Publisher’s note

All claims expressed in this article are solely those of the authors and do not necessarily represent those of their affiliated organizations, or those of the publisher, the editors and the reviewers. Any product that may be evaluated in this article, or claim that may be made by its manufacturer, is not guaranteed or endorsed by the publisher.

## References

[1] World Health Organization. Global recommendations on physical activity for health. Geneva: World Health Organization (2010). 58 p. 26180873

[2] Melville CA Mitchell F Stalker K Matthews L McConnachie A Murray HM. Effectiveness of a walking programme to support adults with intellectual disabilities to increase physical activity: walk well cluster-randomised controlled trial. Int J Behav Nutr Phys Act. (2015) 12:125. doi: 10.1186/s12966-015-0290-5, PMID: 26416606 PMC4587575

[3] Emerson E Baines S. Health inequalities and people with learning disabilities in the UK. Tizard Learn Disabil Rev. (2011) 16:42–8. doi: 10.5042/tldr.2011.0008

[4] Barnett DW Barnett A Nathan A Van Cauwenberg J Cerin E. Built environmental correlates of older adults’ total physical activity and walking: a systematic review and meta-analysis. Int J Behav Nutr Phys Act. (2017) 14:103. doi: 10.1186/s12966-017-0558-z, PMID: 28784183 PMC5547528

[5] Williams PT. Advantage of distance- versus time-based estimates of walking in predicting adiposity. Med Sci Sports Exerc. (2012) 44:1728–37. doi: 10.1249/MSS.0b013e318258af3f, PMID: 22525767 PMC3780575

[6] Jakubec SL Carruthers Den Hoed D Ray H Krishnamurthy A. Mental well-being and quality-of-life benefits of inclusion in nature for adults with disabilities and their caregivers. Landsc Res. (2016) 41:616–27. doi: 10.1080/01426397.2016.1197190

[7] Paterson DH Warburton DER. Physical activity and functional limitations in older adults: a systematic review related to Canada's physical activity guidelines. Int J Behav Nutr Phys Act. (2010) 7:38. doi: 10.1186/1479-5868-7-38, PMID: 20459782 PMC2882898

[8] Tudor-Locke C Craig CL Aoyagi Y Bell RC Croteau KA de Bourdeaudhuij I. How many steps/day are enough? For older adults and special populations. Int J Behav Nutr Phys Act. (2011) 8:80. doi: 10.1186/1479-5868-8-80, PMID: 21798044 PMC3169444

[9] Bull FC Hardman AE. Walking: a best buy for public and planetary health. Br J Sports Med. (2018) 52:755–6. doi: 10.1136/bjsports-2017-098566, PMID: 29187348

[10] Brach JS Vanswearingen JM. Interventions to improve walking in older adults. Curr Transl Geriatr Exp Gerontol Rep. (2013) 2:230–8. doi: 10.1007/s13670-013-0059-0, PMID: 24319641 PMC3851025

[11] Almuhtaseb S Oppewal A Hilgenkamp TIM. Gait characteristics in individuals with intellectual disabilities: a literature review. Res Dev Disabil. (2014) 35:2858–83. doi: 10.1016/j.ridd.2014.07.017 25105568

[12] Uysal SA Erden Z Akbayrak T Demirtürk F. Comparison of balance and gait in visually or hearing impaired children. Percept Mot Skills. (2010) 111:71–80. doi: 10.2466/10.11.15.25.PMS.111.4.71-80 21058587

[13] KN SK Sathish R Vinayak S Pandit TP, Braille assistance system for visually impaired, blind & deaf-mute people in indoor & outdoor application. 2019 4th International conference on recent trends on electronics, information, communication & technology (RTEICT); (2019), 17–18.

[14] Sol ME Verschuren O Horemans H Westers P Visser-Meily JMA De Groot JF. The effects of wheelchair mobility skills and exercise training on physical activity, fitness, skills and confidence in youth using a manual wheelchair. Disabil Rehabil. (2021) 44:4398–407. doi: 10.1080/09638288.2021.1907456 33874820

[15] Kulkarni M Kalantre S Upadhye S Karande S Ahuja S. Approach to learning disability. Indian J Pediatr. (2001) 68:539–46. doi: 10.1007/BF02723250 11450386

[16] Tse ACY. Brief report: impact of a physical exercise intervention on emotion regulation and behavioral functioning in children with autism spectrum disorder. J Autism Dev Disord. (2020) 50:4191–8. doi: 10.1007/s10803-020-04418-2, PMID: 32130593

[17] Ash T Bowling A Davison K Garcia J. Physical activity interventions for children with social, emotional, and behavioral disabilities-a systematic review. J Dev Behav Pediatr. (2017) 38:431–45. doi: 10.1097/DBP.0000000000000452, PMID: 28671892

[18] Pinquart M. Correlates of subjective health in older adults: a meta-analysis. Psychol Aging. (2001) 16:414–26. doi: 10.1037/0882-7974.16.3.414, PMID: 11554520

[19] Jang Y Poon LW Martin P. Individual differences in the effects of disease and disability on depressive symptoms: the role of age and subjective health. Int J Aging Hum Dev. (2004) 59:125–37. doi: 10.2190/RT1W-2HD7-KG5X-K1FB, PMID: 15453141

[20] Baron-Epel O Kaplan G. General subjective health status or age-related subjective health status: does it make a difference? Soc Sci Med. (2001) 53:1373–81. doi: 10.1016/S0277-9536(00)00426-3 11676407

[21] Easterlin RA. Do people adapt to poorer health? Health and health satisfaction over the life cycle In: Maggino F, editor. A life devoted to quality of life: Festschrift in honor of Alex C Michalos. Cham: Springer International Publishing (2016). 81–92.

[22] Michalos AC Zumbo BD. Healthy days, health satisfaction and satisfaction with the overall quality of life. Soc Indic Res. (2002) 59:321–38. doi: 10.1023/A:1019601213926

[23] Heydarian NM Castro Y Morera OF. A brief report of the prevalence of chronic and acute health conditions among blind American adults. Disabil Health J. (2021) 14:101072. doi: 10.1016/j.dhjo.2021.101072, PMID: 33640309 PMC8516085

[24] Canha L Simões C Gaspar Matos M Owens L. Well-being and health in adolescents with disabilities. Psicologia. (2016) 29:32. doi: 10.1186/s41155-016-0041-9

[25] Drum CE Horner-Johnson W Krahn GL. Self-rated health and healthy days: examining the "disability paradox". Disabil Health J. (2008) 1:71–8. doi: 10.1016/j.dhjo.2008.01.002, PMID: 21122714

[26] Lin CY Cheng TC. Health status and life satisfaction among people with disabilities: evidence from Taiwan. Disabil Health J. (2019) 12:249–56. doi: 10.1016/j.dhjo.2018.10.008, PMID: 30409671

[27] Kesavayuth D Rosenman RE Zikos V. Personality and health satisfaction. J Behav Exp Econ. (2015) 54:64–73. doi: 10.1016/j.socec.2014.11.005

[28] Cott CA Gignac MA Badley EM. Determinants of self rated health for Canadians with chronic disease and disability. J Epidemiol Community Health. (1999) 53:731–6. doi: 10.1136/jech.53.11.731, PMID: 10656104 PMC1756802

[29] Crews JE Campbell VA. Vision impairment and hearing loss among community-dwelling older Americans: implications for health and functioning. Am J Public Health. (2004) 94:823–9. doi: 10.2105/AJPH.94.5.823, PMID: 15117707 PMC1448344

[30] Wu S Wang R Zhao Y Ma X Wu M Yan X. The relationship between self-rated health and objective health status: a population-based study. BMC Public Health. (2013) 13:320. doi: 10.1186/1471-2458-13-320, PMID: 23570559 PMC3637052

[31] World Health Organization Regional Office for Europe. Growing up unequal: gender and socioeconomic differences in young People's health and well-being In: Health behaviour in school-aged children (HBSC) study: International report from the 2013/2014 survey. Copenhagen: World Health Organization. Regional Office for Europe (2016). 276.

[32] Lombardo P Jones W Wang L Shen X Goldner EM. The fundamental association between mental health and life satisfaction: results from successive waves of a Canadian national survey. BMC Public Health. (2018) 18:342. doi: 10.1186/s12889-018-5235-x, PMID: 29530010 PMC5848433

[33] Farrar JT Troxel AB Stott C Duncombe P Jensen MP. Validity, reliability, and clinical importance of change in a 0–10 numeric rating scale measure of spasticity: a post hoc analysis of a randomized, double-blind, placebo-controlled trial. Clin Ther. (2008) 30:974–85. doi: 10.1016/j.clinthera.2008.05.011, PMID: 18555944

[34] World Health Organization, World Bank. World report on disability. Geneva: World Health Organization (2011) Report No.: 9789241564182.

[35] Kim WH Park YG Shin HI Im SH. The world report on disability and recent developments in South Korea. Am J Phys Med Rehabil. (2014) 93:S58–62. doi: 10.1097/PHM.0000000000000024, PMID: 24356084

[36] Richards NC Gouda HN Durham J Rampatige R Rodney A Whittaker M. Disability, noncommunicable disease and health information. Bull World Health Organ. (2016) 94:230–2. doi: 10.2471/BLT.15.156869, PMID: 26966336 PMC4773929

[37] Martin Ginis KA van der Ploeg HP Foster C Lai B McBride CB Ng K. Participation of people living with disabilities in physical activity: a global perspective. Lancet. (2021) 398:443–55. doi: 10.1016/S0140-6736(21)01164-8, PMID: 34302764

[38] Bloemen M Van Wely L Mollema J Dallmeijer A de Groot J. Evidence for increasing physical activity in children with physical disabilities: a systematic review. Dev Med Child Neurol. (2017) 59:1004–10. doi: 10.1111/dmcn.13422, PMID: 28374442

[39] Bujang MA Baharum N. Sample size guideline for correlation analysis. World J Soc Sci Res. (2016) 3:37–46. doi: 10.22158/wjssr.v3n1p37

[40] Bujang MA Sa’ata N Joys AR Alic MM. An audit of the statistics and the comparison with the parameter in the population In: The 22nd National Symposium on mathematical sciences (SKSM22). Selangor, Malaysia: AIP Publishing LLC (2015)

[41] Steiber N. Intergenerational educational mobility and health satisfaction across the life course: does the long arm of childhood conditions only become visible later in life? Soc Sci Med. (2019) 242:112603. doi: 10.1016/j.socscimed.2019.112603 31655463

[42] Fritz CO Morris PE Richler JJ. Effect size estimates: current use, calculations, and interpretation. J Exp Psychol Gen. (2012) 141:2–18. doi: 10.1037/a0024338 21823805

[43] Lee C Kim J Yang H. Exploration of life satisfaction of Korean people with sensory impairments across the lifespan. Disabil Health J. (2020) 13:100931. doi: 10.1016/j.dhjo.2020.100931 32327387

[44] Franke KB Hills K Huebner ES Flory K. Life satisfaction in adolescents with autism spectrum disorder. J Autism Dev Disord. (2019) 49:1205–18. doi: 10.1007/s10803-018-3822-4 30443699

[45] Lin L-Y. Quality of life of Taiwanese adults with autism spectrum disorder. PLoS One. (2014) 9:e109567. doi: 10.1371/journal.pone.0109567, PMID: 25299379 PMC4192352

[46] McLeod JD Hawbaker A Meanwell E. The health of college students on the autism spectrum as compared to their neurotypical peers. Autism. (2021) 25:719–30. doi: 10.1177/1362361320926070, PMID: 32551992

[47] Weir E Allison C Baron-Cohen S. Autistic adults have poorer quality healthcare and worse health based on self-report data. Mol Autism. (2022) 13:23. doi: 10.1186/s13229-022-00501-w, PMID: 35619147 PMC9135388

[48] Taggart L Johnston A Mullhall P Hassiotis A Murphy M Slater P. ‘Walk buds’: a walking programme to increase physical activity, physical fitness and emotional wellbeing, in 9–13 yr old children with intellectual disability. A study protocol for a clustered RCT. Contemp Clin Trials. (2022) 119:106856. doi: 10.1016/j.cct.2022.106856, PMID: 35863694

[49] Brooker K Mutch A McPherson L Ware R Lennox N Van Dooren K. “We can talk while we’re walking”: seeking the views of adults with intellectual disability to inform a walking and social-support program. Adapt Phys Act Q. (2015) 32:34–48. doi: 10.1123/apaq.2013-0067 25544719

[50] Garcia-Del Pino-Ramos S Romero-Galisteo RP Pinero-Pinto E Lirio-Romero C Palomo-Carrión R. Effectiveness of treadmill training on the motor development of children with cerebral palsy and down syndrome. Medicina. (2021) 81:367–74. PMID: 34137695

[51] Larsson G Julu POO Witt Engerström I Sandlund M Lindström B. Walking on treadmill with Rett syndrome—effects on the autonomic nervous system. Res Dev Disabil. (2018) 83:99–107. doi: 10.1016/j.ridd.2018.08.010, PMID: 30193160

[52] Bruland D Schulenkorf T Nutsch N Nadolny S Latteck Ä-D. Interventions to improve physical activity in daily life of people with intellectual disabilities. Detailed results presentation of a scoping review. Bielefeld: Universität Bielefeld (2019). 12/01 p.

[53] Stanish HI Temple VA Frey GC. Health-promoting physical activity of adults with mental retardation. Ment Retard Dev Disabil Res Rev. (2006) 12:13–21. doi: 10.1002/mrdd.20090, PMID: 16435324

[54] Kachouri H Laatar R Borji R Rebai H Sahli S. Using a dual-task paradigm to investigate motor and cognitive performance in children with intellectual disability. J Appl Res Intellect Disabil. (2020) 33:172–9. doi: 10.1111/jar.12655, PMID: 31441573

[55] Stanish HI Temple VA. Efficacy of a peer-guided exercise programme for adolescents with intellectual disability. J Appl Res Intellect Disabil. (2012) 25:319–28. doi: 10.1111/j.1468-3148.2011.00668.x, PMID: 22711480

[56] Stanish HI Draheim CC. Walking activity, body composition and blood pressure in adults with intellectual disabilities. J Appl Res Intellect Disabil. (2007) 20:183–90. doi: 10.1111/j.1468-3148.2006.00314.x

[57] Cassady JM. Teachers' attitudes toward the inclusion of students with autism and emotional behavioral disorder. Electronic J Incl Educ. (2011) 2:5.

[58] Ilkım M Tanır H Özdemir M Bozkurt İ. The effect of physical activity on level of anger among individuals with autism. Turk J Sport Exe. (2018) 20:130–5. doi: 10.15314/tsed.446092

[59] Roe J. Cities, green space, and mental well-being. Oxford, UK: Oxford University Press (2016).

[60] Li D Larsen L Yang Y Wang L Zhai Y Sullivan WC. Exposure to nature for children with autism spectrum disorder: benefits, caveats, and barriers. Health Place. (2019) 55:71–9. doi: 10.1016/j.healthplace.2018.11.005, PMID: 30503683

[61] Hall B. Health incentives: the science and art of motivating healthy behaviors. Benefits Q. (2008) 24:12–22. PMID: 18590179

[62] Phoenix C Griffin M Smith B. Physical activity among older people with sight loss: a qualitative research study to inform policy and practice. Public Health. (2015) 129:124–30. doi: 10.1016/j.puhe.2014.10.001, PMID: 25687710

